# Dynamical and Structural Analysis of a T Cell Survival Network Identifies Novel Candidate Therapeutic Targets for Large Granular Lymphocyte Leukemia

**DOI:** 10.1371/journal.pcbi.1002267

**Published:** 2011-11-10

**Authors:** Assieh Saadatpour, Rui-Sheng Wang, Aijun Liao, Xin Liu, Thomas P. Loughran, István Albert, Réka Albert

**Affiliations:** 1 Department of Mathematics, The Pennsylvania State University, University Park, Pennsylvania, United States of America; 2 Department of Physics, The Pennsylvania State University, University Park, Pennsylvania, United States of America; 3 Penn State Hershey Cancer Institute, The Pennsylvania State University College of Medicine, Hershey, Pennsylvania, United States of America; 4 Department of Biochemistry and Molecular Biology, The Pennsylvania State University, University Park, Pennsylvania, United States of America; Bar Ilan University, Israel

## Abstract

The blood cancer T cell large granular lymphocyte (T-LGL) leukemia is a chronic disease characterized by a clonal proliferation of cytotoxic T cells. As no curative therapy is yet known for this disease, identification of potential therapeutic targets is of immense importance. In this paper, we perform a comprehensive dynamical and structural analysis of a network model of this disease. By employing a network reduction technique, we identify the stationary states (fixed points) of the system, representing normal and diseased (T-LGL) behavior, and analyze their precursor states (basins of attraction) using an asynchronous Boolean dynamic framework. This analysis identifies the T-LGL states of 54 components of the network, out of which 36 (67%) are corroborated by previous experimental evidence and the rest are novel predictions. We further test and validate one of these newly identified states experimentally. Specifically, we verify the prediction that the node SMAD is over-active in leukemic T-LGL by demonstrating the predominant phosphorylation of the SMAD family members Smad2 and Smad3. Our systematic perturbation analysis using dynamical and structural methods leads to the identification of 19 potential therapeutic targets, 68% of which are corroborated by experimental evidence. The novel therapeutic targets provide valuable guidance for wet-bench experiments. In addition, we successfully identify two new candidates for engineering long-lived T cells necessary for the delivery of virus and cancer vaccines. Overall, this study provides a bird's-eye-view of the avenues available for identification of therapeutic targets for similar diseases through perturbation of the underlying signal transduction network.

## Author Summary

T-LGL leukemia is a blood cancer characterized by an abnormal increase in the abundance of a type of white blood cell called T cell. Since there is no known curative therapy for this disease, identification of potential therapeutic targets is of utmost importance. Experimental identification of manipulations capable of reversing the disease condition is usually a long, arduous process. Mathematical modeling can aid this process by identifying potential therapeutic interventions. In this work, we carry out a systematic analysis of a network model of T cell survival in T-LGL leukemia to get a deeper insight into the unknown facets of the disease. We identify the T-LGL status of 54 components of the system, out of which 36 (67%) are corroborated by previous experimental evidence and the rest are novel predictions, one of which we validate by follow-up experiments. By deciphering the structure and dynamics of the underlying network, we identify component perturbations that lead to programmed cell death, thereby suggesting several novel candidate therapeutic targets for future experiments.

## Introduction

Living cells perceive and respond to environmental perturbations in order to maintain their functional capabilities, such as growth, survival, and apoptosis. This process is carried out through a cascade of interactions forming complex signaling networks. Dysregulation (abnormal expression or activity) of some components in these signaling networks affects the efficacy of signal transduction and may eventually trigger a transition from the normal physiological state to a dysfunctional system [1] manifested as diseases such as diabetes [2], [3], developmental disorders [4], autoimmunity [5] and cancer [4], [6]. For example, the blood cancer T-cell large granular lymphocyte (T-LGL) leukemia exhibits an abnormal proliferation of mature cytotoxic T lymphocytes (CTLs). Normal CTLs are generated to eliminate cells infected by a virus, but unlike normal CTLs which undergo activation-induced cell death after they successfully fight the virus, leukemic T-LGL cells remain long-term competent [7]. The cause of this abnormal behavior has been identified as dysregulation of a few components of the signal transduction network responsible for activation-induced cell death in T cells [8].

Network representation, wherein the system's components are denoted as nodes and their interactions as edges, provides a powerful tool for analyzing many complex systems [9], [10], [11]. In particular, network modeling has recently found ever-increasing applications in understanding the dynamic behavior of intracellular biological systems in response to environmental stimuli and internal perturbations [12], [13], [14]. The paucity of knowledge on the biochemical kinetic parameters required for continuous models has called for alternative dynamic approaches. Among the most successful approaches are discrete dynamic models in which each component is assumed to have a finite number of qualitative states, and the regulatory interactions are described by logical functions [15]. The simplest discrete dynamic models are the so-called Boolean models that assume only two states (ON or OFF) for each component. These models were originally introduced by S. Kauffman and R. Thomas to provide a coarse-grained description of gene regulatory networks [16], [17].

**Figure 1:**
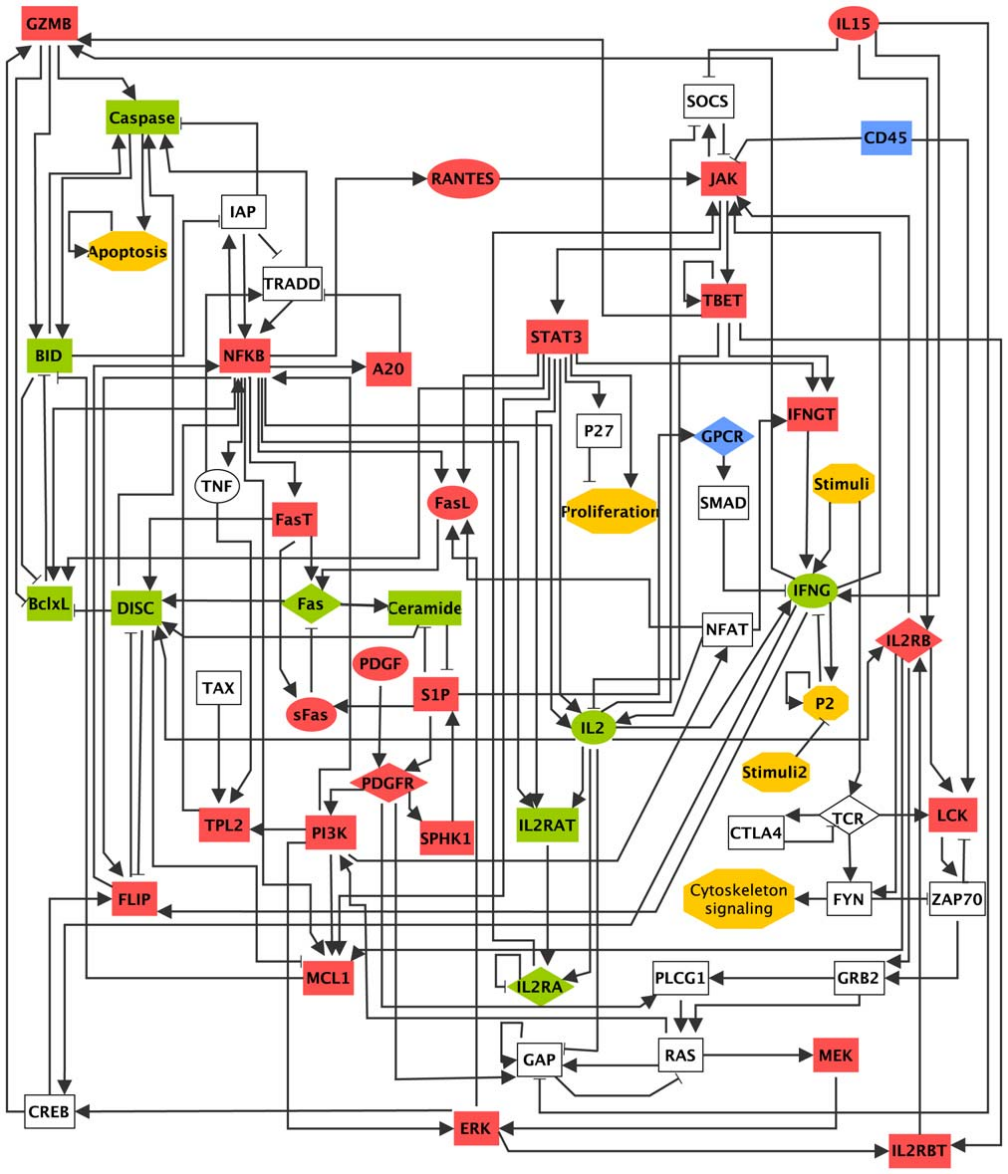
The T-LGL survival signaling network. The shape of the nodes indicates the cellular location: rectangular indicates intracellular components, ellipse indicates extracellular components, and diamond indicates receptors. Node colors reflect the current knowledge on the state of these nodes in leukemic cells: highly active components in T-LGL are shown in red, inhibited nodes are shown in green, nodes that have been suggested to be deregulated are in blue, and the state of white nodes is unknown. Conceptual nodes (Stimuli, Stimuli2, P2, Cytoskeleton signaling, Proliferation, and Apoptosis) are represented by yellow hexagons. An arrowhead or a short perpendicular bar at the end of an edge indicates activation or inhibition, respectively. The inhibitory edges from Apoptosis to other nodes are not shown. The full names of the node labels are given in Table S2. This figure and its caption are adapted from [18].

A Boolean network model of T cell survival signaling in the context of T-LGL leukemia was previously constructed by Zhang *et al* [18] through performing an extensive literature search. This network consists of 60 components, including proteins, mRNAs, and small molecules (see Figure 1). The main input to the network is “Stimuli”, which represents virus or antigen stimulation, and the main output node is “Apoptosis”, which denotes programmed cell death. Based on a random order asynchronous Boolean dynamic model of the assembled network, Zhang *et al* identified a minimal number of dysregulations that can cause the T-LGL survival state, namely overabundance or overactivity of the proteins platelet-derived growth factor (PDGF) and interleukin 15 (IL15). Zhang *et al* carried out a preliminary analysis of the network's dynamics by performing numerical simulations starting from one specific initial condition (corresponding to resting T cells receiving antigen stimulation and over-abundance of the two proteins PDGF and IL15). Once the known deregulations in T-LGL leukemia were reproduced, each of these deregulations was interrupted individually, by setting the node's status to the opposite state, to predict key mediators of the disease. Yet, a complete dynamic analysis of the system, including identification of the attractors (e.g. steady states) of the system and their corresponding basin of attraction (precursor states), as well as a thorough perturbation analysis of the system considering all possible initial states, is lacking. Performing this analysis can provide deeper insights into unknown aspects of T-LGL leukemia.

Stuck-at-ON/OFF fault is a very common dysregulation of biomolecules in various cancer diseases [19]. For example, stuck-at-ON (constitutive activation) of the RAS protein in the mitogen-activated protein kinase pathways leads to aberrant cell proliferation and cancer [19], [20]. Thus identifying components whose stuck-at values result in the clearance, or alternatively, the persistence of a disease is extremely beneficial for the design of intervention strategies. As there is no known curative therapy for T-LGL leukemia, identification of potential therapeutic targets is of utmost importance [21].

In this paper, we carry out a detailed analysis of the T-LGL signaling network by considering all possible initial states to probe the long-term behavior of the underlying disease. We employ an asynchronous Boolean dynamic framework and a network reduction method, which we previously proposed [22], to identify the attractors of the system and analyze their basins of attraction. This analysis allows us to confirm or predict the T-LGL states of 54 components of the network. The predicted state of one of the components (SMAD) is validated by new wet-bench experiments. We then perform node perturbation analysis using the dynamic approach and a structural method proposed in [23] to study to what extent does each component contribute to T-LGL leukemia. Both methods give consistent results and together identify 19 key components whose disruption can reverse the abnormal state of the signaling network, thereby uncovering potential therapeutic targets for this disease, some of which are also corroborated by experimental evidence.

## Materials and Methods

Any biological regulatory network can be represented by a directed graph *G* = (*V*, *E*) where *V* = {*v*_1_, *v*_2_,…, *v_n_*} is the set of vertices (nodes) describing different components of the system, and *E* is the set of edges denoting the regulatory interactions among the components. The orientation of each edge in the network follows the direction of mass transfer or information propagation from the upstream to the downstream node. Each edge can be also characterized with a sign where a positive sign denotes activation and a negative sign signifies inhibition. The source nodes (i.e. nodes with no incoming edges) of this graph, if they exist, represent external inputs (signals), and one or more nodes, usually sink nodes (i.e. nodes with no outgoing edges), are customarily designated as outputs of the network.

### Boolean dynamic models

Boolean models belong to the class of discrete dynamic models in which each node of the network is characterized by an ON (1) or OFF (0) state and usually the time variable *t* is also considered to be discrete, i.e. it takes nonnegative integer values [24], [25]. The future state of each node *v_i_* is determined by the current states of the nodes regulating it according to a Boolean transfer function 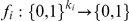, where *k_i_* is the number of regulators of *v_i_*. Each Boolean function (rule) represents the regulatory relationships between the components and is usually expressed via the logical operators AND, OR and NOT. The state of the system at each time step is denoted by a vector whose *i* ^th^ component represents the state of node *v_i_* at that time step. The discrete state space of a system can be represented by a state transition graph whose nodes are states of the system and edges are allowed transitions among the states. By updating the nodes' states at each time step, the state of the system evolves over time and following a trajectory of states it eventually settles down into an attractor. An attractor can be in the form of either a fixed point, in which the state of the system does not change, or a complex attractor, where the system oscillates (regularly or irregularly) among a set of states. The set of states leading to a specific attractor is called the basin of attraction of that attractor.

In order to evaluate the state of each node at a given time instant, synchronous as well as asynchronous updating strategies have been proposed [24], [25]. In the synchronous method all nodes of the network are updated simultaneously at multiples of a common time step. The underlying assumption of this update method is that the timescales of all the processes occurring in a system are similar. This is a quite strong and potentially unrealistic assumption, which in particular may not be suited for intracellular biological processes due to the variety of timescales associated with transcription, translation and post-translational mechanisms [26]. To overcome this limitation, various asynchronous methods have been proposed wherein the nodes are updated based on individual timescales [25], [27], [28], [29], [30], including deterministic methods with fixed node timescales and stochastic methods such as random order asynchronous method [27] wherein the nodes are updated in random permutations. In a previous work [22], we carried out a comparative study of three different asynchronous methods applied to the same biological system. That study suggested that the general asynchronous (GA) method, wherein a randomly selected node is updated at each time step, is the most efficient and informative asynchronous updating strategy. This is because deterministic asynchronous [22] or autonomous [30] Boolean models require kinetic or timing knowledge, which is usually missing, and random order asynchronous models [27] are not computationally efficient compared to the GA models. In addition, the superiority of the GA approach has been corroborated by other researchers [29] and the method has been used in other studies as well [31], [32]. We thus chose to employ the GA method in this work, and we implemented it using the open-source software library BooleanNet [33]. It is important to note that the stochasticity inherent to this method may cause each state to have multiple successors, and thus the basins of attraction of different attractors may overlap. For systems with multiple fixed-point attractors, the absorption probabilities to each fixed point can be computed through the analysis of the Markov chain and transition matrix associated with the state transition graph of the system [34]. Given a fixed point, node perturbations can be performed by reversing the state of the nodes i.e. by knocking out the nodes that stabilize in an ON state in the fixed point or over-expressing the ones that stabilize in an OFF state.

### Network reduction

A Boolean network with *n* nodes has a total of 2*^n^* states. This exponential dependence makes it computationally intractable to map the state transition graphs of even relatively small networks. This calls for developing efficient network reduction approaches. Recent efforts towards addressing this challenge consists of iteratively removing single nodes that do not regulate their own function and simplifying the redundant transfer functions using Boolean algebra [35], [36]. Naldi *et al* [35] proved that this approach preserves the fixed points of the system and that for each (irregular) complex attractor in the original asynchronous model there is at least one complex attractor in the reduced model (i.e. network reduction may create spurious oscillations). Boolean networks often contain nodes whose states stabilize in an attracting state after a transient period, regardless of updating strategy or initial conditions. The attracting states of these nodes can be readily identified by inspection of their Boolean functions. In a previous work [22] we proposed a method of network simplification by (*i*) pinpointing and eliminating these stabilized nodes and (*ii*) iteratively removing a simple mediator node (e.g. a node that has one incoming edge and one outgoing edge) and connecting its input(s) to its target(s). Our simplification method shares similarities with the method proposed in [35], [36], with the difference that we only remove stabilized nodes (which have the same state on every attractor) and simple mediator nodes rather than eliminating each node without a self loop. Thus their proof regarding the preservation of the steady states by the reduction method holds true in our case. We employed this simplification method for the analysis of a signal transduction network in plants and verified by using numerical simulations that it preserves the attractors of that system. In this work, we employ this reduction method to simplify the T-LGL leukemia signal transduction network synthesized by Zhang *et al* [18], thereby facilitating its dynamical analysis. We also note that the first step of our simplification method is similar to the logical steady state analysis implemented in the software tool CellNetAnalyzer [37], [38]. We thus refer to this step as logical steady state analysis throughout the paper.

### Identification of attractors

It should be noted that the fixed points of a Boolean network are the same for both synchronous and asynchronous methods. In order to obtain the fixed points of a system one can solve the set of Boolean equations independent of time. To this end, we first fix the state of the source nodes. We then determine the nodes whose rules depend on the source nodes and will either stabilize in an attracting state after a time delay or otherwise their rules can be simplified significantly by plugging in the state of the source nodes. Iteratively inserting the states of stabilized nodes in the rules (i.e. employing logical steady state analysis) will result in either the fixed point(s) of the system, or the partial fixed point(s) and a remaining set of equations to be solved. In the latter case, if the remaining set of equations is too large to obtain its fixed point(s) analytically, we take advantage of the second step of our reduction method [22] to simplify the resulting network and to determine a simpler set of Boolean rules. By solving this simpler set of equations (or performing numerical simulations, if necessary) and plugging the solutions into the original rules, we can then find the states of the removed nodes and determine the attractors of the whole system accordingly. For the analysis of basins of attraction of the attractors, we perform numerical simulations using the GA update method.

### A structural method for identifying essential components

The topology (structure) and the function of biological networks are closely related. Therefore, structural analysis of biological networks provides an alternative way to understand their function [39], [40]. We have recently proposed an integrative method to identify the essential components of any given signal transduction network [23]. The starting point of the method is to represent the combinatorial relationship of multiple regulatory interactions converging on a node *v* by a Boolean rule:
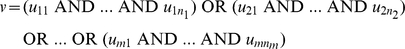
where *u*_ij_'s are regulators of node *v*. The method consists of two main steps. The first step is the expansion of a signaling network to a new representation by incorporating the sign of the interactions as well as the combinatorial nature of multiple converging interactions. This is achieved by introducing a complementary node for each component that plays a role in negative regulations (NOT operation) as well as introducing a composite node to denote conditionality among two or more edges (AND operation). This step eliminates the distinction of the edge signs; that is, all directed edges in the expanded network denote activation. In addition, the AND and OR operators can be readily distinguished in the expanded network, i.e., multiple edges ending at composite nodes are added by the AND operator, while multiple edges ending at original or complementary nodes are cumulated by the OR operator. The second step is to model the cascading effects following the loss of a node by an iterative process that identifies and removes nodes that have lost their indispensable regulators. These two steps allow ranking of the nodes by the effects of their loss on the connectivity between the network's input(s) and output(s). We proposed two connectivity measures in [23], namely the simple path (SP) measure, which counts the number of all simple paths from inputs to outputs, and a graph measure based on elementary signaling modes (ESMs), defined as a minimal set of components that can perform signal transduction from initial signals to cellular responses. We found that the combinatorial aspects of ESMs pose a substantial obstacle to counting them in large networks and that the SP measure has a similar performance as the ESM measure since both measures incorporate the cascading effects of a node's removal arising from the synergistic relations between multiple interactions. Therefore, we employ the SP measure and define the importance value of a component *v* as:
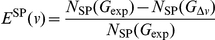
where * N*_SP_(*G*_exp_) and * N*_SP_(*G*_Δ*v*_) denote the total number of simple paths from the input(s) to the output(s) in the original expanded network *G*_exp_ and the damaged network *G*_Δ*v*_ upon disruption of node *v*, respectively. This essentiality measure takes values in the interval [0,1], with 1 indicating a node whose loss causes the disruption of all paths between the input and output node(s). In this paper, we also make use of this structural method to identify essential components of the T-LGL leukemia signaling network. We then relate the importance value of nodes to the effects of their knockout (sustained OFF state) in the dynamic model and the importance value of complementary nodes to the effects of their original nodes' constitutive activation (sustained ON state) in the dynamic model.

### Experimental determination of the T-LGL state of the node SMAD

#### Patient characteristics and preparation of peripheral blood mononuclear cells (PBMC)

All patients met the clinical criteria of T-LGL leukemia with increased numbers (>80%) of CD3^+^CD8^+^ T cells in the peripheral blood. Patients received no treatment at the time of sample acquisition. Peripheral blood specimens from LGL leukemia patients were obtained and informed consents signed for sample collection according to a protocol approved by the Institutional Review Board of Penn State Hershey Cancer Institute. PBMC were isolated by Ficoll-Hypaque gradient separation, as described previously [41]. CD3^+^CD8^+^ T cells from four age- and gender-matched healthy donors were isolated by a human CD8^+^ T cell enrichment cocktail RosetteSep kit (Stemcell Technology). The purity of freshly isolated CD3^+^CD8^+^ T cells (2×10^5^/sample in triplicate) in each of the samples was determined by flow cytometry assay by detecting positive staining of the CD3 and CD8 T cell markers. The purity for normal purified CD3^+^CD8^+^ T cells was over 90%. Cell viability was determined by trypan blue exclusion assay with more than 95% viability in all the samples.

#### Phospho-Smad2 and phospho-Smad3 measurement

Western blot was performed to detect Phospho-Smad2 (P-Smad2) and Phospho-Smad3 (P-Smad3) in activated normal CD3^+^CD8^+^ cells (CD3^+^CD8^+^ cells >90%) compared with PBMC (CD3^+^CD8^+^ cells >80%) from T-LGL leukemia patients. Normal CD3^+^CD8^+^ T cells were isolated by a human CD8^+^ T cell enrichment cocktail RosetteSep kit (Stemcell Technology) from four normal donors, then cultured in RPMI-1640 supplemented with 10% fetal bovine serum in presence of PHA (1 µg/mL) for 1 day followed by IL2 (500 IU/mL) for 3 days (lanes 1–4). The equal loading of protein was confirmed by probing with total Smad2 or Smad3. Phospho-Smad2 (Ser465/467), Smad2, Phospho-Smad3 (Ser423/425) and Smad3 antibodies were purchased from Cell Signaling Technology Inc. (Beverly, MA).

## Results

### Network simplification and dynamic analysis

The T-LGL signaling network reconstructed by Zhang *et al* [18] contains 60 nodes and 142 regulatory edges. Zhang *et al* used a two-step process: they first synthesized a network containing 128 nodes and 287 edges by extensive literature search, then simplified it with the software NET-SYNTHESIS [42], which constructs the sparsest network that maintains all of the causal (upstream-downstream) effects incorporated in a redundant starting network. In this study, we work with the 60-node T-LGL signaling network reported in [18], which is redrawn in Figure 1. The Boolean rules for the components of the network were constructed in [18] by synthesizing experimental observations and for convenience are given in Table S1 as well. The description of the node names and abbreviations are provided in Table S2.

**Figure 2:**
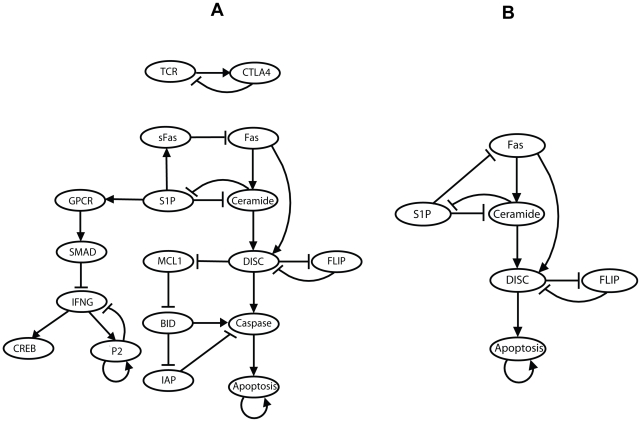
Reduced sub-networks of the T-LGL signaling network. The full names of the nodes can be found in Table S2. An arrowhead or a short perpendicular bar at the end of an edge indicates activation or inhibition, respectively. The inhibitory edges from Apoptosis to other nodes are not shown. **(A) The 18-node sub-network.** This sub-network is obtained by removing the nodes that stabilize in the ON or OFF state upon fixing the state of the source nodes. **(B) The 6-node sub-network.** This sub-network is obtained by removing the top sub-graph of the sub-network in (A) and merging simple mediator nodes in the bottom sub-graph.

To reduce the computational burden associated with the large state space (more than 10^18^ states for 60 nodes), we simplified the T-LGL network using the reduction method proposed in [22] (see Materials and Methods). We fixed the six source nodes in the states given in [18], i.e. Stimuli, IL15, and PDGF were fixed at ON and Stimuli2, CD45, and TAX were fixed at OFF. We used the Boolean rules constructed in [18], with one notable difference. The Boolean rules for all the nodes in [18], except Apoptosis, contain the expression “AND NOT Apoptosis”, meaning that if Apoptosis is ON, the cell dies and correspondingly all other nodes are turned OFF. To focus on the trajectory leading to the initial turning on of the Apoptosis node, we removed the “AND NOT Apoptosis” from all the logical rules. This allows us to determine the stationary states of the nodes in a live cell. We determined which nodes' states stabilize using the first step of our simplification method, i.e. logical steady state analysis (see Materials and Methods). Our analysis revealed that 36 nodes of the network stabilize in either an ON or OFF state. In particular, Proliferation and Cytoskeleton signaling, two output nodes of the network, stabilize in the OFF and ON state, respectively. Low proliferation in leukemic LGL has been observed experimentally [43], which supports our finding of a long-term OFF state for this output node. The ON state of Cytoskeleton signaling may not be biologically relevant as this node represents the ability of T cells to attach and move which is expected to be reduced in leukemic T-LGL compared to normal T cells. The nodes whose stabilized states cannot be readily obtained by inspection of their Boolean rules form the sub-network represented in Figure 2A. The Boolean rules of these nodes are listed in Table S3 wherein we put back the “AND NOT Apoptosis” expression into the rules.

**Figure 3:**
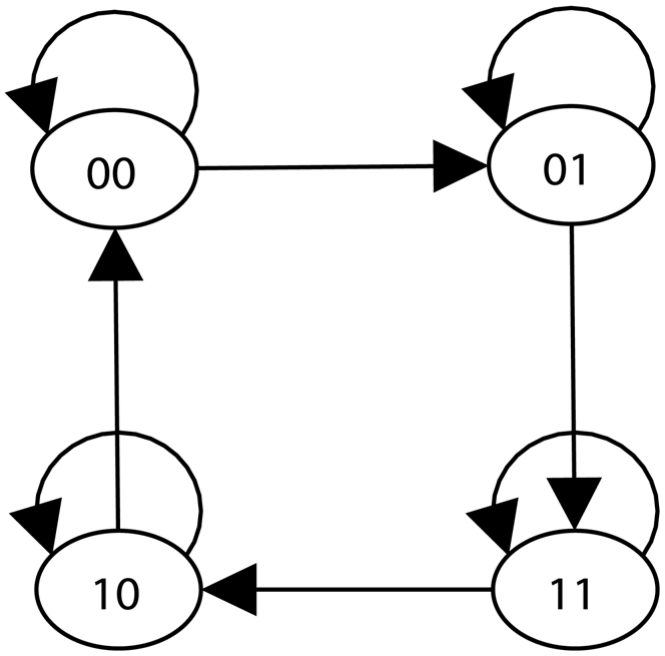
The state transition graph corresponding to the two oscillatory nodes, CTLA4 and TCR. In this graph the left binary digit of the node identifier indicates the state of CTLA4 and the right digit represents the state of TCR. The directed edges represent state transitions allowed by updating a single node's state; self-loops appear when a node is updated but its state does not change.

Next, we identified the attractors (long-term behavior) of the sub-network represented in Figure 2A (see Materials and Methods). We found that upon activation of Apoptosis all other nodes stabilize at OFF, forming the normal fixed point of the system, which represents the normal behavior of programmed cell death. When Apoptosis is stabilized at OFF, the two nodes in the top sub-graph oscillate while all the nodes in the bottom sub-graph are stabilized at either ON or OFF. As shown in Figure 3, the state space of the two oscillatory nodes, TCR and CTLA4, forms a complex attractor in which the average fraction of ON states for either node is 0.5. Given that these two nodes have no effect on any other node under the conditions studied here (i.e. stable states of the source nodes), their behavior can be separated from the rest of the network.

The bottom sub-graph exhibits the normal fixed point, as well as two T-LGL (disease) fixed points in which Apoptosis is OFF. The only difference between the two T-LGL fixed points is that the node P2 is ON in one fixed point and OFF in the other, which was expected due to the presence of a self-loop on P2 in Figure 2A. P2 is a virtual node introduced to mediate the inhibition of interferon-γ translation in the case of sustained activity of the interferon-γ protein (IFNG in Figure 2A). The node IFNG is also inhibited by the node SMAD which stabilizes in the ON state in both T-LGL fixed points. Therefore IFNG stabilizes at OFF, irrespective of the state of P2, as supported by experimental evidence [44]. Thus the biological difference between the two fixed points is essentially a memory effect, i.e. the ON state of P2 indicates that IFNG was transiently ON before stabilizing in the OFF state. In the two T-LGL fixed points for the bottom sub-graph of Figure 2A, the nodes sFas, GPCR, S1P, SMAD, MCL1, FLIP, and IAP are ON and the other nodes are OFF. We found by numerical simulations using the GA method (see Materials and Methods) that out of 65,536 total states in the state transition graph, 53% are in the exclusive basin of attraction of the normal fixed point, 0.24% are in the exclusive basin of attraction of the T-LGL fixed point wherein P2 is ON and 0.03% are in the exclusive basin of attraction of the T-LGL fixed point wherein P2 is OFF. Interestingly, there is a significant overlap among the basins of attraction of all the three fixed points. The large basin of attraction of the normal fixed point is partly due to the fact that all the states having Apoptosis in the ON state (that is, half of the total number of states) belong to the exclusive basin of the normal fixed point. These states are not biologically relevant initial conditions but they represent potential intermediary states toward programmed cell death and as such they need to be included in the state transition graph.

Since the state transition graph of the bottom sub-graph given in Figure 2A is too large to represent and to further analyze (e.g. to obtain the probabilities of reaching each of the fixed points), we applied the second step of the network reduction method proposed in [22]. This step preserves the fixed points of the system (see Materials and Methods), and since the only attractors of this sub-graph are fixed points, the state space of the reduced network is expected to reflect the properties of the full state space. Correspondingly, the nodes having in-degree and out-degree of one (or less) in the sub-graph on Figure 2A, such as sFas, MCL1, IAP, GPCR, SMAD, and CREB, can be safely removed without losing any significant information as such nodes at most introduce a delay in the signal propagation. In addition, we note that although the node P2 has a self-loop and generates a new T-LGL fixed point as described before, it can also be removed from the network since the two fixed points differ only in the state of P2 and thus correspond to biologically equivalent disease states. We revisit this node when enumerating the attractors of the original network. In the resulting simplified network, the nodes BID, Caspase, and IFNG would also have in-degree and out-degree of one (or less) and thus can be safely removed as well. This reduction procedure results in a simple sub-network represented in Figure 2B with the Boolean rules given in Table 1.

**Table 1:** Boolean rules governing the nodes' states in the 6-node sub-network represented in Figure 2B.

Node	Boolean rule
S1P	S1P* = NOT (Ceramide OR Apoptosis)
FLIP	FLIP* = NOT (DISC OR Apoptosis)
Fas	Fas* = NOT (S1P OR Apoptosis)
Ceramide	Ceramide* = Fas AND NOT (S1P OR Apoptosis)
DISC	DISC* = (Ceramide OR (Fas AND NOT FLIP)) AND NOT Apoptosis
Apoptosis	Apoptosis* = DISC OR Apoptosis

For simplicity, the nodes' states are represented by the node names. The symbol * indicates the future state of the marked node.

Our attractor analysis revealed that this sub-network has two fixed points, namely 000001 and 110000 (the digits from left to right represent the state of the nodes in the order as listed from top to bottom in Table 1). The first fixed point represents the normal state, that is, the apoptosis of CTL cells. Note that the OFF state of other nodes in this fixed point was expected because of the presence of “AND NOT Apoptosis” in all the Boolean rules. The second fixed point is the T-LGL (disease) one as Apoptosis is stabilized in the OFF state. We note that the sub-network depicted in Figure 2B contains a backbone of activations from Fas to Apoptosis and two nodes (S1P and FLIP) which both have a mutual inhibitory relationship with the backbone. If activation reaches Apoptosis, the system converges to the normal fixed point. In the T-LGL fixed point, on the other hand, the backbone is inactive while S1P and FLIP are active.

**Figure 4:**
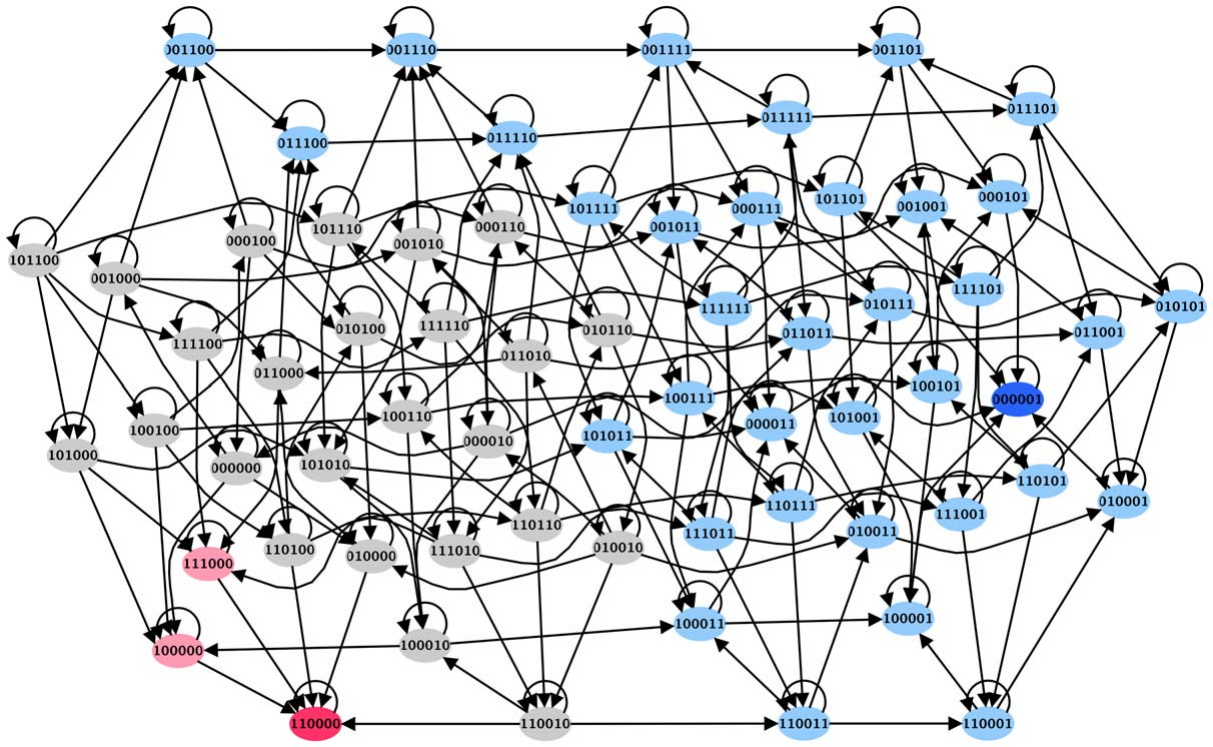
The state transition graph of the 6-node sub-network represented in **Figure 2B**. It contains 64 states of which the state shown with a dark blue symbol is the normal fixed point and the state shown in red is the T-LGL fixed point. States denoted by light blue symbols are uniquely in the basin of attraction of the normal fixed point whereas the states in pink can only reach the T-LGL fixed point. Gray states, on the other hand, can lead to either fixed point.

**Figure 5:**
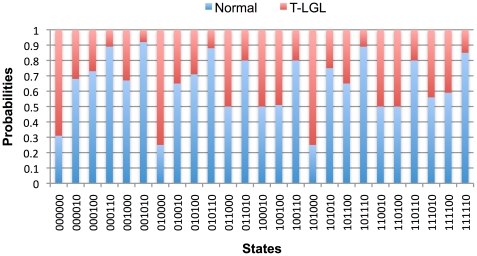
The probabilities of reaching the normal and T-LGL fixed points when both are reachable. These probabilities are computed starting from the states that are shared by both basins of attraction (see gray-colored states illustrated in Figure 4).

We found by simulations that for the simplified network of Figure 2B, 56% of the states of the state transition graph (represented in Figure 4) are in the exclusive basin of attraction of the normal fixed point while 5% of the states form the exclusive basin of attraction of the T-LGL fixed point. Again, the half of state space that has the ON state of Apoptosis belongs to the exclusive basin of attraction of the normal fixed point. Notably, there is a significant overlap between the basins of attraction of the two fixed points, which is illustrated by a gray color in Figure 4. The probabilities of reaching each of the two fixed points starting from these gray-colored states, found by analysis of the corresponding Markov chain (see Materials and Methods), are given in Figure 5. As this figure represents, for the majority of cases the probability of reaching the normal fixed point is higher than that of the T-LGL fixed point. The three states whose probabilities to reach the T-LGL fixed point are greater than or equal to 0.7 are one step away either from the T-LGL fixed point or from the states in its exclusive basin of attraction. In two of them, the backbone of the network in Figure 2B is inactive, and in the third one the backbone is partially inactive and most likely will remain inactive due to the ON state of S1P (one of the two nodes having mutual inhibition with the backbone).

Based on the sub-network analysis and considering the states of the nodes that stabilized at the beginning based on the logical steady state analysis, we conclude that the whole T-LGL network has three attractors, namely the normal fixed point wherein Apoptosis is ON and all other nodes are OFF, representing the normal physiological state, and two T-LGL attractors in which all nodes except two, i.e. TCR and CTLA4, are in a steady state, representing the disease state. These T-LGL attractors are given in the second column of Table 2, which presents the predicted T-LGL states of 54 components of the network (all but the six source nodes whose state is indicated at the beginning of the Results section). We note that the two T-LGL attractors essentially represent the same disease state since they only differ in the state of the virtual node P2. Moreover, this disease state can be considered as a fixed point since only two nodes oscillate in the T-LGL attractors. For this reason we will refer to this state as the T-LGL fixed point. It is expected that the basins of attraction of the fixed points have similar features as those of the simplified networks.

**Table 2:** A summary of the dynamic analysis results of the T-LGL survival signaling network.

Node	T-LGL state	Ref.	Fixed point the disruption leads to	Size of exclusive basin of normal fixed point	Ref.
DISC	OFF	[46]	Normal	100%	[46]
Ceramide	OFF	[48]	Normal	100%	[48]
Caspase	OFF	[46]	Normal	100%	
SPHK1	ON	[21]	Normal	100%	[18]
S1P	ON	[21]	Normal	100%	[21]
PDGFR	ON	[59]	Normal	100%	[18]
GAP	OFF*		Normal	100%	
RAS	ON*		Normal	100%	[41] 1
MEK	ON	[59]	Normal	100%	[41] 1
ERK	ON	[50], [59]	Normal	100%	[41] 1
IL2RBT	ON	[60]	Normal	100%	
IL2RB	ON	[60]	Normal	100%	
STAT3	ON	[49]	Normal	100%	[49]
BID	OFF	[45]	Normal	100%	
MCL1	ON	[49]	Normal	100%	[49]
SOCS	OFF*		Both	81%	
JAK	ON	[49]	Both	81%	[49]
PI3K	ON	[50]	Both	75%	[50]
NFκB	ON	[18]	Both	75%	[18]
Fas	OFF	[48]	Both	72%	
sFas	ON	[61]	Both	72%	
TBET	ON	[18]	Both	63%	
RANTES	ON	[44]	Both	63%	
PLCG1	ON*		Both	63%	
FLIP	ON	[46]	Both	56%	
IL2	OFF	[62]	Both	56%	
IAP	ON*		Both	56%	
TNF	ON*		Both	56%	
BclxL	OFF	[49]	Both	56%	
GZMB	ON	[63]	Both	56%	
IL2RA	OFF	[62]	Both	56%	
NFAT	ON*		Both	56%	
GRB2	ON*		Both	56%	
IFNGT	ON	[44], [62]	Both	56%	
TRADD	OFF*		Both	56%	
ZAP70	OFF*		Both	56%	
LCK	ON	[50]	Both	56%	
FYN	ON*		Both	56%	
IFNG	OFF	[44]	Both	56%	
SMAD	ON*	This study	Both	56%	
GPCR	ON	[21], [64]	Both	56%	
TPL2	ON	[65]	Both	56%	
A20	ON	[21]	Both	56%	
IL2RAT	OFF	[62]	Both	56%	
CREB	OFF*		Both	56%	
P27	ON*		Both	56%	
P2	ON/OFF		Both	56%	
FasT	ON	[48]	T-LGL	0%	
FasL	ON	[48]	T-LGL	0%	
Cytoskeleton signaling	ON*		—	—	
Proliferation	OFF	[43]	—	—	
Apoptosis	OFF	[66]	—	—	
TCR	Oscillate*		—	—	
CTLA4	Oscillate*		—	—	

The first two columns from the left list the components of the network (except for the six source nodes) and their T-LGL states. The nodes' states marked with a * symbol were not documented experimentally in T-LGL before and were predicted by our steady state analysis. The references for the nodes' states documented before are given in the third column. The fixed point(s) obtained after each of the nodes' states is reversed is given in the fourth column, while the size of the exclusive basin of attraction of the normal fixed point, expressed as a percentage of the whole relevant state space, is indicated in the fifth column. The reference of the perturbation cases for which experimental evidence exists is given in the last column. The first 19 nodes in the first column are potential therapeutic targets for T-LGL leukemia.

1 Evidence in NK-LGL leukemia.

### Experimental validation of the T-LGL steady state

Experimental evidence exists for the deregulated states of 36 (67%) components out of the 54 predicted T-LGL states as summarized in the third column of Table 2. For example, the stable ON state of MEK, ERK, JAK, and STAT3 indicates that the MAPK and JAK-STAT pathways are activated. The OFF state of BID is corroborated by recent evidence that it is down-regulated both in natural killer (NK) and in T cell LGL leukemia [45]. In addition, the node RAS was found to be constitutively active in NK-LGL leukemia [41], which indirectly supports our result on the predicted ON state of this node. For three other components, namely, GPCR, DISC, and IFNG, which were classified as being deregulated without clear evidence of either up-regulation or down-regulation in [18], we found that they eventually stabilize at ON, OFF, and OFF, respectively. The OFF state of IFNG and DISC is indeed supported by experimental evidence [44], [46]. In the second column of Table 2, we indicated with an asterisk the stabilized state of 17 components that were experimentally undocumented before and thus are predictions of our steady state analysis (P2 was not included as it is a virtual node). We note that ten of these cases were also predicted in [18] by simulations.

**Figure 6:**
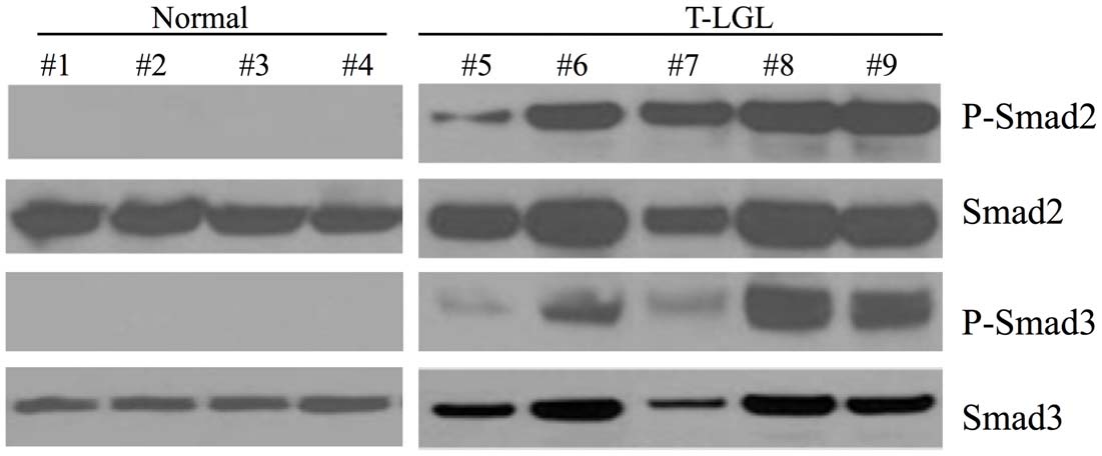
Experimental validation of the increased activity (ON state) of Smad2/3 in leukemic T-LGL. Western blot detection of phosphorylated Smad2 or Smad3, and total Smad2 (i.e. the sum of phosphorylated and non-phosphorylated Smad2) or Smad3 in activated normal T cells compared with peripheral blood mononuclear cells from T-LGL leukemia patients confirms that Smad2 or Smad3 is unphosphorylated (inactive) in normal T cells and predominantly phosphorylated (active) in T-LGL cells.

The predicted T-LGL states of these 17 components can guide targeted experimental follow-up studies. As an example of this approach, we tested the predicted over-activity of the node SMAD (see Materials and Methods). As described in [18] the SMAD node represents a merger of SMAD family members Smad 2, 3, and 4. Smad 2 and 3 are receptor-regulated signaling proteins which are phosphorylated and activated by type I receptor kinases while Smad4 is an unregulated co-mediator [47]. Phosphorylated Smad2 and/or Smad3 form heterotrimeric complexes with Smad4 and these complexes translocate to the nucleus and regulate gene expression. Thus an ON state of SMAD in the model is a representation of the predominance of phosphorylated Smad2 and/or phosphorylated Smad3 in T-LGL cells. In relative terms as compared to normal (resting or activated) T cells, the predicted ON state implies a higher level of phosphorylated Smad2/3 in T-LGL cells as compared to normal T cells. Indeed, as shown in Figure 6, T cells of T-LGL patients tend to have high levels of phosphorylated Smad2/3, while normal activated T cells have essentially no phosphorylated Smad2/3. Thus our experiments validate the theoretical prediction.

### Node perturbations

A question of immense biological importance is which manipulations of the T-LGL network can result in consistent activation-induced cell death and the elimination of the dysregulated (diseased) behavior. We can rephrase and specify this question as which node perturbations (knockouts or constitutive activations) lead to a system that has only the normal fixed point. These perturbations can serve as candidates for potential therapeutic interventions. To this end, we performed node perturbation analysis using both structural and dynamic methods.

#### Structural perturbation analysis

For the structural analysis, using the T-LGL network (Figure 1) and the Boolean rules (Table S1), we constructed an expanded T-LGL survival signaling network (see Materials and Methods) as represented in Figure S1. In order to evaluate the importance of signaling components mediating T-LGL leukemia, we introduced the complementary node of Apoptosis (denoted by ∼Apoptosis in Figure S1) as an output representing the survival of the CTL cells, which is activated by the complementary node of Caspase (denoted by ∼Caspase in Figure S1). The reason is that we are interested in the question of how to make this outcome (i.e., the disease state) disappear, or in graph terminology, disconnected from the inputs of the network. In order to count all the simple paths from a single (rather than multiple) input signal to the output node, we fixed the states of Stimuli and IL15 at ON and those of Stimuli2, CD45, and TAX at OFF. Once the Boolean rules were simplified, we determined all the signaling paths from PDGF to the output node ∼Apoptosis. Interestingly, we found that the number of signaling paths from PDGF to ∼Apoptosis is much smaller than the number of signaling paths from PDGF to Apoptosis (78,827 versus 346,974), consistent with the finding from dynamic analysis that the exclusive basin of attraction of the T-LGL fixed point is much smaller than that of the normal fixed point.

**Figure 7:**
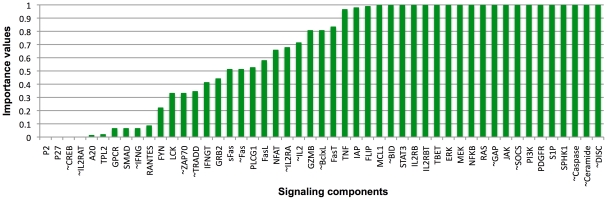
Importance values of network components in T-LGL leukemia. These values are based on the relative reduction of the number of paths from PDGF to ∼Apoptosis after considering the cascading effects of node disruptions. The complementary nodes are denoted by the corresponding original nodes with a symbol ‘∼’ as prefix representing ‘negation’.

Our goal of identifying node state manipulations that lead to the apoptosis of the abnormally surviving T-LGL cells can be translated into the graph-theoretical problem of finding key nodes that mediate paths to the node ∼Apoptosis. Elimination of these nodes has the potential to make ∼Apoptosis unreachable, or in other words to make Apoptosis the only reachable outcome. The T-LGL fixed point determined in dynamic analysis serves as a list of candidate deletions. Accordingly, we separately deleted each node that stabilizes at ON in the T-LGL fixed point, and each complementary node whose corresponding original node stabilizes at OFF in the T-LGL fixed point (see Table 2 for the state of nodes in the T-LGL fixed point). We then calculated the importance values of these nodes by examining the cascading effects of their deletion on the number of simple paths from PDGF to the ∼Apoptosis output (see Materials and Methods). The importance values of the signaling components are given in Figure 7. As we can see in this figure, several components, including ∼DISC, ∼Ceramide, ∼Caspase, SPHK1, S1P, PDGFR, PI3K, ∼SOCS, JAK, ∼GAP, RAS, NFκB, MEK, and ERK have importance values of one (or very close to one). This means that blocking any of these nodes disrupts (almost) all signaling paths from the source node to ∼Apoptosis, thus these nodes are candidate therapeutic targets.

#### Dynamic perturbation analysis

To identify manipulations of the T-LGL network leading to the existence of only the normal fixed point, we first considered the following scenario. We assumed that the T-LGL network is the simplified network given in Figure 2B. We examined the following dynamic perturbation approaches as potential interventions propelling the system into the normal fixed point. In the first two approaches, it is assumed that the T-LGL fixed point has been already reached (i.e. the disease has already developed), and in the last approach, all possible initial conditions are considered.

1. Reverse the state of one node at a time in the T-LGL fixed point for only the first time step, and keep updating the system. This intervention may be accomplished by a pharmacological intervention on a T-LGL cell.
2. Reverse the state of one node in the T-LGL fixed point permanently and continue updating other nodes. This intervention may be accomplished by genetic engineering of a T-LGL cell.
3. Considering all possible initial states, fix the state of one node in the opposite of its T-LGL state and keep updating other nodes. This intervention may be accomplished by genetic engineering of a population of CTLs.

For the first perturbation approach, we found that only the trivial case of flipping the state of Apoptosis to ON leads exclusively to the normal fixed point. Using the second perturbation approach, we observed that fixing S1P at OFF or Apoptosis at ON eliminates the T-LGL fixed point. In addition, fixing either Ceramide or DISC at ON results in a new fixed point which is similar to the normal fixed point of the unperturbed system, with the only difference that the disrupted node's state is fixed at ON as long as the cell is alive. Using the last perturbation approach, we found a result identical to that of the second approach, indicating that the nodes S1P, Ceramide, and DISC are candidate therapeutic targets for the simplified sub-network. Experiments also confirm that Ceramide and DISC can serve as therapeutic targets [46], [48]. We note that the third approach is superior to the second in that it provides additional information on the size of the basin of attraction of each fixed point. For example, we observed that in the case of over-expression of Fas, the exclusive basin of attraction of the normal fixed point increases significantly to 72% of the states. This suggests that although both fixed points are still reachable, the normal fixed point is more probable to be reached. This analysis revealed that the last approach leads to more detailed results than the first two approaches.

Next we focused our attention to the effects of node disruptions on the whole network to make biologically testable predictions about the occurrence of the disease state under different conditions. To this end, we followed the third approach delineated above. More precisely, for each node disruption we fixed the state of that node in the opposite of its stabilized state in the T-LGL fixed point given in Table 2 (i.e. we knocked out the nodes that stabilize in the ON state in T-LGL fixed point and over-expressed the ones that stabilize in the OFF state) and considered all possible initial states for the remaining nodes (except for the six source nodes). Of the 60 nodes of the network, six are source nodes, three are output nodes and two (CTLA4 and TCR) have oscillatory behavior in the T-LGL attractor. For each of the remaining nodes, we fixed the state of that node in the opposite of its T-LGL state, initiated the six source nodes as in the unperturbed case, and identified the stabilized nodes using logical steady state analysis (see Materials and Methods). We then simplified the network of non-stabilized nodes according to the second step of our reduction method (see Materials and Methods) and obtained all possible fixed points by solving the corresponding set of Boolean equations. For some cases we needed to construct the full state transition graphs because of the possibility of oscillation (e.g. when the two oscillatory nodes, CTLA4 and TCR, were connected to other nodes in the simplified network and there was a possibility of propagating the oscillation to other nodes in the T-LGL state). We found that in the case of perturbation of TBET, PI3K, NFκB, JAK, or SOCS, five additional nodes of the network connected to CTLA4 and TCR, namely LCK, FYN, Cytoskeleton signaling, ZAP70, and GRB2, oscillate as well. Also, for the knockout of FYN, only two of these additional nodes, i.e. LCK and ZAP70 oscillate. In addition, in the case of perturbation of TBET, JAK, SOCS, or IL2, the node IL2RA shows oscillatory behavior in the T-LGL state.

In general, two types of fixed points were observed, the normal fixed point with Apoptosis being ON and all other nodes being OFF, and similar-to-TLGL fixed points with Apoptosis being OFF and the state of some nodes being different from the wild-type T-LGL fixed point due to the disruption imposed on the network. We still consider these latter fixed points as the T-LGL fixed point. A summary of the node disruption results, including the fixed point(s) obtained after the disruption as well as the size of the exclusive basin of attraction of the normal fixed point in the respective reduced model, is given in the fourth and fifth columns of Table 2. Our results indicate that disruption of any of the first 15 nodes in Table 2 leads to the disappearance of the T-LGL fixed point (i.e., of the disease state). These nodes are thus predicted candidate therapeutic targets. For example, our results suggest that knockout of STAT3 or over-expression of Ceramide in deregulated CTLs restores their activation induced cell death. We found for the knockout of either FasT or FasL that the normal fixed point and the 50% of the state transition graph which includes the ON state of Apoptosis is separated from the rest of the state space and thus they are not accessible from the biologically relevant initial conditions. Therefore, the T-LGL fixed point is the only biologically relevant outcome in this case. For this reason, the size of the basin of attraction of the normal fixed point was indicated as 0% in Table 2. Notably, these nodes can serve as candidates for engineering of long-lived T cells, which are necessary for the delivery of virus and cancer vaccines. The remaining node disruptions still retain both disease and normal fixed points.

There is corroborating literature evidence for several of the therapeutic targets predicted by our analysis. For example, it was found experimentally that STAT3 knockdown by using siRNA or down-regulation of MCL1 through inhibiting STAT3 induces apoptosis in leukemic T-LGL [49]. Furthermore, *in vitro* Ceramide treatment induces apoptosis in leukemic T-LGL [48]. It was also found that treatment with IL2 and TCR stimulation facilitates Fas-mediated apoptosis via induction of DISC formation [46]. In addition, SPHK1 inhibition by using chemical inhibitors significantly induces apoptosis in leukemic T-LGL [18]. These experimental results validate that perturbation of these nodes results in the normal fixed point as mentioned in Table 2. Moreover, it was reported in [41] that inhibition of RAS through introducing a dominant negative form of RAS, or inhibition of MEK or ERK through chemical inhibitors, induces apoptosis in leukemic NK-LGL, which indirectly supports our results on these three nodes.

For the cases where both fixed points are still reachable, our analysis of the relative size of the basins of attraction (i.e. percentage of the whole relevant state space) of the fixed points and the probabilities of reaching the fixed points (see Materials and Methods) indicated that in most of these cases the trends are similar to the wild-type model, e.g. the size of the exclusive basin of attraction of the normal fixed point is 56%, the same as that for the unperturbed system. In a few cases, however, including JAK, PI3K, or NFκB knockout as well as SOCS over-expression, the exclusive basin of attraction of the normal fixed point increased significantly (to 75% or more). Thus, these nodes can be also considered as potential therapeutic targets. Interestingly, for three cases, namely JAK, PI3K, and NFκB, experimental data also suggest that the balance between the incidence of the two fixed points is shifted in the manipulated system compared to the original one. For example, inhibition of JAK [49], PI3K [50] or NFκB [18] through chemical inhibitors induces apoptosis in leukemic T-LGL. In summary, our analysis leads to the novel predictions that Caspase, GAP, BID, or SOCS over-expression as well as RAS, MEK, ERK, IL2RBT, or IL2RB knockout can lead to apoptosis of T-LGL cells.

#### Comparison between structural and dynamic perturbation analysis

**Figure 8:**
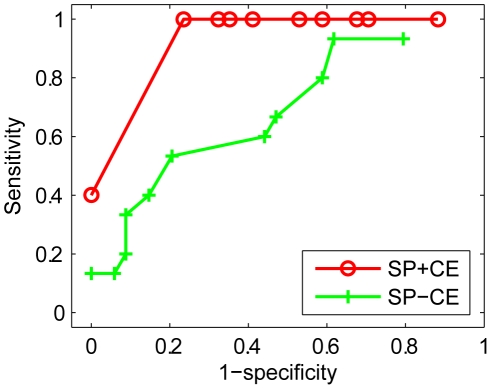
Comparison of structural perturbation analysis results with and without cascading effects of node deletions. SP+CE represents the simple path measure considering cascading effects of node deletions, and SP-CE represents the simple path measure without considering cascading effects of node deletions.

We performed the perturbation analysis using a dynamic method as well as a structural method. How do the results compare? From the dynamic analysis, a node is classified as an important mediator of the T-LGL fixed point if reversing its state from the value it achieves in the T-LGL fixed point will lead the system to have only the normal fixed point. From the structural analysis, a node can be classified as an important mediator of the T-LGL behavior if its importance value (see Materials and Methods) to the ∼Apoptosis outcome is higher than a pre-specified threshold. We used different importance values as thresholds and compared the structure-based classification with the dynamics-based classification by using the latter as the standard. The sensitivity (the fraction of important components based on dynamic perturbation analysis that are recognized as important by the structural method) and specificity (the fraction of non-important components based on dynamic perturbation analysis that are recognized as non-important by the structural method) values of the structure-based classification are summarized by the red curve in Figure 8. The structural method gives the best fit to the dynamic method (namely, sensitivity of 1.00 and specificity of 0.76) if a threshold of 0.9 is used. An important feature of the structural method is its incorporation of the cascading effects of a node's deletion. To illustrate this point, we also show the corresponding result without considering the cascading effects of nodes' deletions represented by the green curve in Figure 8. As this figure demonstrates, the results using a pure topological measure without considering the cascading effects gives a much worse fit to the results of the dynamic method.

Interestingly, for all the components whose manipulation lead the system to have only the normal fixed point according to the dynamic analysis (the first 15 components in Table 2), the reported importance values based on the structural method were larger than 0.95. For four additional cases, namely, SOCS, JAK, PI3K, and NFκB, which are identified as important for survival based on the simple path measure, the dynamic analysis results also revealed that the T-LGL outcome has a lesser probability to be reached as mentioned earlier. Therefore, they can also be considered as potential therapeutic targets.

We note that there are four cases, namely, TBET, FLIP, IAP, and TNF, which were identified as important based on the structural method while their disruption maintains the existence of both fixed points based on dynamic analysis and the size of the exclusive basin of attraction of the normal fixed point is either close to or the same as that of the wild-type system. This may be partly due to the fact that in the state space analysis we consider all possible initial conditions for the system, whereas the topological analysis implicitly refers to only one initial condition, wherein three source nodes are ON and all other nodes are OFF. Another potential reason regarding the discrepancies between the structural and dynamic perturbation results might be related to the structural method's use of the simple path measure rather than the elementary signaling modes (ESMs, see Materials and Methods). Furthermore, although the reduction method used for the dynamic analysis preserves the fixed points, it can change the state transition graph and thus may have an impact on the relative size of the basins of attraction, serving as an alternative source of inconsistencies. However, this change is not expected to be drastic as we found that the exclusive basin of attraction of the normal fixed point in the 6-node network was approximately of the same relative size as that in the 18-node network.

## Discussion

In this paper we presented a comprehensive analysis of the T-LGL survival signaling network to unravel the unknown facets of this disease. By using a reduction technique, we first identified the fixed points of the system, namely the normal and T-LGL fixed points, which represent the healthy and disease states, respectively. This analysis identified the T-LGL states of 54 components of the network, out of which 36 (67%) are corroborated by previous experimental evidence and the rest are novel predictions. These new predictions include RAS, PLCG1, IAP, TNF, NFAT, GRB2, FYN, SMAD, P27, and Cytoskeleton signaling, which are predicted to stabilize at ON in T-LGL leukemia and GAP, SOCS, TRADD, ZAP70, and CREB which are predicted to stabilize at OFF. In addition, we found that the node P2 can stabilize in either the ON or OFF state, whereas two nodes, TCR and CTLA4, oscillate. We have experimentally validated the prediction that the node SMAD is over-active in leukemic T-LGL by demonstrating the predominant phosphorylation of the SMAD family members Smad2 and Smad3. The predicted T-LGL states of other nodes provide valuable guidance for targeted experimental follow-up studies of T-LGL leukemia.

Among the predicted states, the ON state of Cytoskeleton signaling may not be biologically relevant as this node represents the ability of T cells to attach and move which is expected to be reduced in leukemic T-LGL compared to normal T cells. This discrepancy may be due to the fact that the network contains insufficient detail regarding the regulation of the cytoskeleton, as there is only one node, FYN, upstream of Cytoskeleton signaling in the network. While the network is able to successfully capture survival signaling without necessarily capturing the cytoskeleton signaling, this discrepancy suggests that follow-up experimental studies should be conducted to determine the relationship between cytoskeleton signaling and survival signaling in the T-LGL network. We note that in the case of perturbation of TBET, PI3K, NFκB, JAK, or SOCS, the node Cytoskeleton signaling exhibits oscillatory behavior induced by oscillations in TCR. At present it is not known whether this predicted behavior is relevant.

Using the general asynchronous (GA) Boolean dynamic approach, we analyzed the basins of attraction of the fixed points. We found that the basin of attraction of the normal fixed point is larger than that of the T-LGL fixed point. The trajectories starting from each initial state toward the T-LGL fixed point (Figure 4) may be indicative of the accumulating deregulations that lead to the disease-associated stable survival state. Although the fixed points, being time independent, are the same for all update methods or implementations of time, the update method may affect the structure of the state transition graph of the system and the basins of attraction of the fixed points. We note that the GA method assumes that each node has an equal chance of being updated. If quantitative or kinetic information becomes available in this system, unequal probabilities may be implemented by grouping the nodes into several “priority classes” and assigning a weight to each class where higher weights indicate more probable transitions [51]. Incorporating such information into the state space may prune the allowed trajectories and give further insights into the accumulation of deregulations.

We took one step further by performing a perturbation analysis using dynamical and structural methods to identify the interventions leading to the disappearance of the disease fixed point. We note that our study has a dramatically larger scope than the previous key mediator analysis of Zhang *et al* [18]. For the dynamical analysis, we employed the GA approach instead of the random order asynchronous method and considered all possible initial conditions as opposed to performing numerical simulations using a specific initial condition. Zhang *et al* only focused on the node Apoptosis, and identified as “key mediators” the nodes whose altered state increases the frequency of ON state of Apoptosis. An increase in Apoptosis' ON state does not necessarily imply that apoptosis is the only possible final outcome of the system. In this work, after finding the fixed points, which completely describe the state of the whole system, we performed dynamic perturbation analysis by fixing the state of each node to its opposite state in the T-LGL fixed point and determining which fixed points were obtained and what their basins of attraction were. This way we were able to identify and distinguish the key mediators whose altered state completely eliminates the leukemic outcome, and those whose altered state reduces the basin of attraction of the leukemic outcome. Moreover, numerical simulations, as done in [18], may not be able to thoroughly sample different timing. In this study, using a reduction technique, we found the cases when timing does not matter with certainty (where there is only one fixed point), and also the cases in which timing and initial conditions may matter (where there are two reachable fixed points). For the perturbation analysis using the structural method, we used the simple path (SP) measure to identify important mediators of the disease outcome and observed consistent results with the dynamic analysis. Our dynamical and structural analysis led to the identification of 19 therapeutic targets (the first 19 nodes in the first column of Table 2), 53% of which are supported by direct experimental evidence and 15% of which are supported by indirect evidence.

Multi-stability (having multiple steady states) is an intrinsic dynamic property of many disease networks [52], [53], which is related to the presence of feedback loops in the network. In a graph-theoretical sense, a feedback loop is a directed cycle whose sign depends upon the parity of the number of negative interactions in the cycle. A positive/negative feedback loop has an even/odd number of negative interactions. It was conjectured that the presence of positive feedback loops in the network is necessary for multi-stability whereas the existence of negative feedback loops is required for having sustained oscillations [54]. From a biological point of view, the former dynamical property is associated with multiple cell types after differentiation while the latter is related to stable periodic behaviors such as circadian rhythms [55]. We note that the T-LGL signaling network consists of both positive and negative feedbacks and thus has a potential for both multi-stability and oscillations. Indeed, the negative feedback in the top sub-graph of Figure 2A causes the complex attractor shown in Figure 3. In contrast, the negative feedback on the node P2 of the bottom sub-graph is counteracted by the positive self-loop on the same node, thus no complex attractor is possible for the bottom sub-graph of Figure 2A. The two mutual inhibition-type positive feedback loops present in the bottom sub-graph and the self-loop on P2 generate the three fixed points, while the positive self-loop on Apoptosis maintains the normal fixed point once Apoptosis is turned ON.

Negative feedback loops can be a source of oscillations [56], homeostasis [56], or excitation-adaptation behavior [57]. Especially, when the activation is slower than the inhibitory interaction in the negative feedback, it can lead to sustained oscillations [56]. In the T-LGL network, the negative feedback loop between the T cell receptor TCR and CTLA4 modulates stimulus-induced activation of the receptor in such a way that CTLA4 is indirectly activated after prolonged TCR activation, whereas the inhibition of TCR by CTLA4 is a direct interaction [58]. That is, activation is slower than inhibition in the negative feedback and thus an oscillatory behavior reminiscent of that obtained by our asynchronous Boolean model would also be observed in continuous modeling frameworks as well. Although no time-measurements of the T cell receptor activity in T-LGL exist, it has been reported that there is variability for TCR activation in different patients ([43] and unpublished observation by T.P. Loughran), supporting the absence of a steady state behavior.

Our study revealed that both structural and dynamic analysis methods can be employed to identify therapeutic targets of a disease, however, they differ in implementation efficiency as well as the scope and applicability of the results. The structural analysis does not require mapping of the state space and thus is less computationally intensive and is more feasible for large network analysis, but it may not capture all the initial states and thus may miss or inaccurately identify some important features. The dynamic analysis method, while computationally intensive, yields a comprehensive picture of the state transition graph, including all possible fixed points of the system, their corresponding basins of attraction, as well as the relative frequency of trajectories leading to each fixed point. We demonstrated that the limitations related to the vast state space of large networks can be overcome by judicious use of the network reduction technique that we developed in our previous study [22]. We conclude that the structural method incorporating the cascading effects of node disruptions is best employed for quick exploratory analysis, and dynamic analysis should be performed to get a thorough and detailed insight into the behavior of a system. Overall, the combined analysis presented in this study opens a promising avenue to predict dysregulated components and identify potential therapeutic targets, and it is versatile enough to be successfully applied to a large variety of signal transduction and regulatory networks related to diseases.

## Supporting Information

Figure S1 **The expanded T-LGL survival signaling network.** Composite nodes are represented by small gray solid circles, original nodes are represented by large ovals, and complementary nodes are represented by rectangles. The labels of complementary nodes are denoted by the labels for the corresponding original nodes with a symbol ‘∼’ as prefix representing ‘negation’.
(TIF)

Table S1 **Boolean rules governing the state of the T-LGL signaling network depicted in** **Figure 1** **.** For simplicity, the nodes' states are represented by the node names. The symbol * indicates the future state of the marked node. The Boolean rule for each node is determined based on the nature of interactions between that node and the nodes directly interacting with it. This rule can be expressed using the logical operators AND, OR and NOT. For example, if the given node has a single upstream node, the corresponding Boolean function would include only one variable. This variable will be combined with a NOT operator if the upstream node is an inhibitor. In cases where the given node has multiple upstream nodes, their effect is combined with AND or OR operators (potentially in conjunction with the NOT operator) to correctly recast the regulatory interactions. For example, the AND operator is used when the co-expression of two (or more) activating inputs is required for activating the target node, whereas, the OR operator implies that the activity of at least one of the upstream activators is sufficient to activate the target node. The type of each interaction (i.e. the logical rule) should be extracted from the relevant literature and experimental evidence. This table is adapted from [1]. The interested reader is referred to [1] for the detailed explanation of the rules.
(PDF)

Table S2 **The full names of components in the T-LGL signaling network corresponding to the abbreviated node labels used in** **Figure 1** **.** Several network nodes represent the union of a few proteins with similar roles. In such cases, a single entry in the first column corresponds to several entries in the second column. This table and its caption are adapted from [1].
(PDF)

Table S3 **Boolean rules governing the state of the 18-node sub-network depicted in** **Figure 2A** **.** For simplicity, the nodes' states are represented by the node names. The symbol * indicates the future state of the marked node.
(PDF)
