# Supplementary figures and images for: Dynamical and Structural Analysis of a T Cell Survival Network Identifies Novel Candidate Therapeutic Targets for Large Granular Lymphocyte Leukemia
Source: PLoS Comput Biol. 2011 Nov 10;7(11):e1002267. doi: 10.1371/journal.pcbi.1002267 (PMC3213185; doi:10.1371/journal.pcbi.1002267)

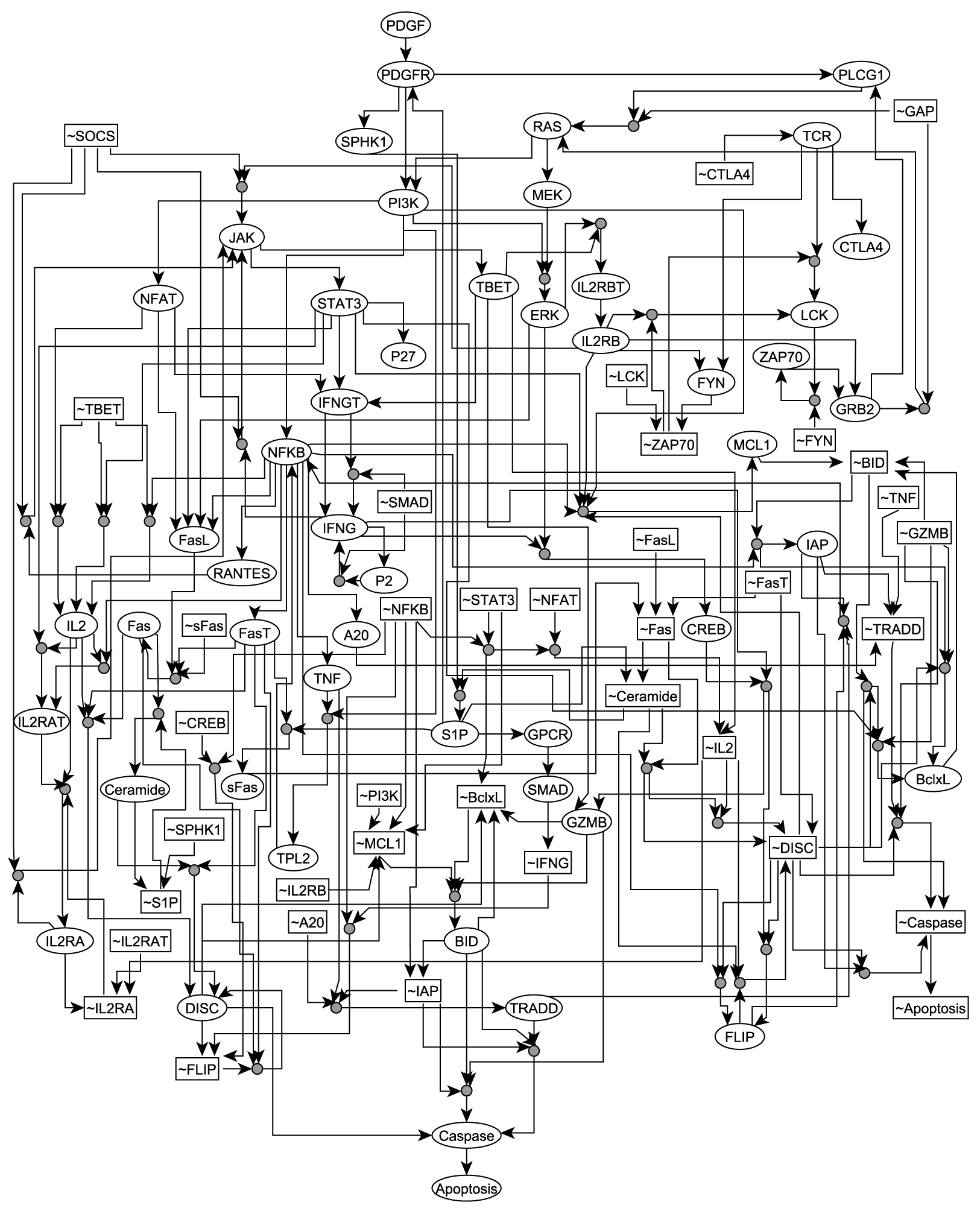

Supplement: Figure S1 — The expanded T-LGL survival signaling network. Composite nodes are represented by small gray solid circles, original nodes are represented by large ovals, and complementary nodes are represented by rectangles. The labels of complementary nodes are denoted by the labels for the corresponding original nodes with a symbol ‘∼’ as prefix representing ‘negation’. (TIF) [file pcbi.1002267.s001.tif]
