# Supplementary material for: Dynamical and Structural Analysis of a T Cell Survival Network Identifies Novel Candidate Therapeutic Targets for Large Granular Lymphocyte Leukemia
Source: PLoS Comput Biol. 2011 Nov 10;7(11):e1002267. doi: 10.1371/journal.pcbi.1002267 (PMC3213185; doi:10.1371/journal.pcbi.1002267)
Supplement: Table S1 — Boolean rules governing the state of the T-LGL signaling network depicted in Figure 1 . For simplicity, the nodes' states are represented by the node names. The symbol * indicates the future state of the marked node. The Boolean rule for each node is determined based on the nature of interactions between that node and the nodes directly interacting with it. This rule can be expressed using the logical operators AND, OR and NOT. For example, if the given node has a single upstream node, the corresponding Boolean function would include only one variable. This variable will be combined with a NOT operator if the upstream node is an inhibitor. In cases where the given node has multiple upstream nodes, their effect is combined with AND or OR operators (potentially in conjunction with the NOT operator) to correctly recast the regulatory interactions. For example, the AND operator is used when the co-expression of two (or more) activating inputs is required for activating the target node, whereas, the OR operator implies that the activity of at least one of the upstream activators is sufficient to activate the target node. The type of each interaction (i.e. the logical rule) should be extracted from the relevant literature and experimental evidence. This table is adapted from [1]. The interested reader is referred to [1] for the detailed explanation of the rules. (PDF) [file pcbi.1002267.s002.pdf]

Table S1. Boolean rules governing the state of the T-LGL signaling network depicted in Figure 1. For simplicity, the nodes' states are represented by the node names. The symbol * indicates the future state of the marked node. The Boolean rule for each node is determined based on the nature of interactions between that node and the nodes directly interacting with it. This rule can be expressed using the logical operators AND, OR and NOT. For example, if the given node has a single upstream node, the corresponding Boolean function would include only one variable. This variable will be combined with a NOT operator if the upstream node is an inhibitor. In cases where the given node has multiple upstream nodes, their effect is combined with AND or OR operators (potentially in conjunction with the NOT operator) to correctly recast the regulatory interactions. For example, the AND operator is used when the co-expression of two (or more) activating inputs is required for activating the target node, whereas, the OR operator implies that the activity of at least one of the upstream activators is sufficient to activate the target node. The type of each interaction (i.e. the logical rule) should be extracted from the relevant literature and experimental evidence. This table is adapted from [1]. The interested reader is referred to [1] for the detailed explanation of the rules.

CTLA4* = TCR AND NOT Apoptosis
TCR* = Stimuli AND NOT (CTLA4 OR Apoptosis)
PDGFR* = (S1P OR PDGF) AND NOT Apoptosis
FYN* = (TCR OR IL2RB) AND NOT Apoptosis
Cytoskeleton signaling* = FYN AND NOT Apoptosis
LCK* = (CD45 OR ((TCR OR IL2RB) AND NOT ZAP70)) AND NOT Apoptosis
ZAP70* = LCK AND NOT (FYN OR Apoptosis)
GRB2* = (IL2RB OR ZAP70) AND NOT Apoptosis
PLCG1* = (GRB2 OR PDGFR) AND NOT Apoptosis
RAS* = (GRB2 OR PLCG1) AND NOT (GAP OR Apoptosis)
GAP* = (RAS OR (PDGFR AND GAP)) AND NOT (IL15 OR IL2 OR Apoptosis)
MEK* = RAS AND NOT Apoptosis
ERK* = (MEK AND PI3K) AND NOT Apoptosis
PI3K* = (PDGFR OR RAS) AND NOT Apoptosis
NFκB* = ((TPL2 OR PI3K) OR (FLIP AND TRADD AND IAP)) AND NOT Apoptosis
NFAT* = PI3K AND NOT Apoptosis
RANTES* = NFκB AND NOT Apoptosis
IL2* = (NFκB OR STAT3 OR NFAT) AND NOT (TBET OR Apoptosis)
IL2RBT* = (ERK AND TBET) AND NOT Apoptosis
IL2RB* = (IL2RBT AND (IL2 OR IL15)) AND NOT Apoptosis
IL2RAT* = (IL2 AND (STAT3 OR NFκB)) AND NOT Apoptosis
IL2RA* = (IL2 AND IL2RAT) AND NOT (IL2RA OR Apoptosis)
JAK* = (IL2RA OR IL2RB OR RANTES OR IFNG) AND NOT (SOCS OR CD45 OR Apoptosis)
SOCS* = JAK AND NOT (IL2 OR IL15 OR Apoptosis)
STAT3* = JAK AND NOT Apoptosis
P27* = STAT3 AND NOT Apoptosis
Proliferation* = STAT3 AND NOT (P27 OR Apoptosis)
TBET* = (JAK OR TBET) AND NOT Apoptosis
CREB* = (ERK AND IFNG) AND NOT Apoptosis
IFNGT* = (TBET OR STAT3 OR NFAT) AND NOT Apoptosis
IFNG* = ((IL2 OR IL15 OR Stimuli) AND IFNGT) AND NOT (SMAD OR P2 OR Apoptosis)
P2* = (IFNG OR P2) AND NOT (Stimuli2 OR Apoptosis)
GZMB* = ((CREB AND IFNG) OR TBET) AND NOT Apoptosis
TPL2* = (TAX OR (PI3K AND TNF)) AND NOT Apoptosis
TNF* = NFκB AND NOT Apoptosis
TRADD* = TNF AND NOT (IAP OR A20 OR Apoptosis)
FasL* = (STAT3 OR NFκB OR NFAT OR ERK) AND NOT Apoptosis
FasT* = NFκB AND NOT Apoptosis

Fas* = (FasT AND FasL) AND NOT (sFas OR Apoptosis)
sFas*[1] = FasT AND S1P AND NOT Apoptosis
Ceramide* = Fas AND NOT (S1P OR Apoptosis)
DISC* = (FasT AND ((Fas AND IL2) OR Ceramide OR (Fas AND NOT FLIP))) AND NOT Apoptosis
Caspase* = ((((TRADD OR GZMB) AND BID) AND NOT IAP) OR DISC) AND NOT Apoptosis
FLIP* = (NFκB OR (CREB AND IFNG)) AND NOT (DISC OR Apoptosis)
A20* = NFκB AND NOT Apoptosis
BID* = (Caspase OR GZMB) AND NOT (BclxL OR MCL1 OR Apoptosis)
IAP* = NFκB AND NOT (BID OR Apoptosis)
BclxL* = (NFκB OR STAT3) AND NOT (BID OR GZMB OR DISC OR Apoptosis)
MCL1*[1] = (IL2RB AND STAT3 AND NFκB AND PI3K) AND NOT (DISC OR Apoptosis)
Apoptosis* = Caspase OR Apoptosis
GPCR* = S1P AND NOT Apoptosis
SMAD* = GPCR AND NOT Apoptosis
SPHK1* = PDGFR AND NOT Apoptosis
S1P* = SPHK1 AND NOT (Ceramide OR Apoptosis)

[1]The rules for these two nodes were updated following personal communication with R. Zhang.

Reference

1. Zhang R, Shah MV, Yang J, Nyland SB, Liu X, et al. (2008) Network model of survival signaling in large granular lymphocyte leukemia. Proc Natl Acad Sci U S A 105: 16308-16313.
