# Supplementary material for: Dynamical and Structural Analysis of a T Cell Survival Network Identifies Novel Candidate Therapeutic Targets for Large Granular Lymphocyte Leukemia
Source: PLoS Comput Biol. 2011 Nov 10;7(11):e1002267. doi: 10.1371/journal.pcbi.1002267 (PMC3213185; doi:10.1371/journal.pcbi.1002267)
Supplement: Table S2 — The full names of components in the T-LGL signaling network corresponding to the abbreviated node labels used in Figure 1 . Several network nodes represent the union of a few proteins with similar roles. In such cases, a single entry in the first column corresponds to several entries in the second column. This table and its caption are adapted from [1]. (PDF) [file pcbi.1002267.s003.pdf]

Table S2. The full names of components in the T-LGL signaling network corresponding to the abbreviated node labels used in Figure 1. Several network nodes represent the union of a few proteins with similar roles. In such cases, a single entry in the first column corresponds to several entries in the second column. This table and its caption are adapted from [1].

**Abbreviated node name**	**Full name**
PDGF	Platelet-derived growth factor beta polypeptide (simian sarcoma viral (v-sis) oncogene homolog)
IL15	Interleukin 15
Stimuli	Antigen stimulation
Stimuli2	New or stronger antigen stimulation
CD45	Protein tyrosine phosphatase, receptor type, C
TAX	Tax p40 (Human T-lymphotropic virus 1)
CTLA4	Cytotoxic T-lymphocyte-associated protein 4
TCR	TCR receptor and coreceptors in CD8 cells
PDGFR	Platelet-derived growth factor receptor, alpha polypeptide<br>Platelet-derived growth factor receptor, beta polypeptide
FYN	FYN oncogene related to SRC, FGR, YES
Cytoskeleton signaling	N/A
LCK	Lymphocyte-specific protein tyrosine kinase
ZAP70	Zeta-chain (TCR) associated protein kinase 70kDa
GRB2	Growth factor receptor-bound protein 2
PLCG1	Phospholipase C, gamma 1
RAS	v-Ha-ras Harvey rat sarcoma viral oncogene homolog
GAP	RAS p21 protein activator (GTPase activating protein) 1
MEK	Mitogen-activated protein kinase kinase 1<br>Mitogen-activated protein kinase kinase 2
ERK	Mitogen-activated protein kinase 1<br>Mitogen-activated protein kinase 3
PI3K	Phosphoinositide-3-kinase, catalytic, alpha polypeptide<br>Phosphoinositide-3-kinase, catalytic, beta polypeptide<br>Phosphoinositide-3-kinase, catalytic, gamma polypeptide
NFκB	Nuclear factor of kappa light polypeptide gene enhancer in B-cells 1 (p105)<br>Nuclear factor of kappa light polypeptide gene enhancer in B-cells 2 (p49/p100)<br>v-rel reticuloendotheliosis viral oncogene homolog A, nuclear factor of kappa light polypeptide gene enhancer in B-cells 3, p65 (avian)
NFAT	Nuclear factor of activated T cells, cytoplasmic, calcineurin-dependent 1<br>Nuclear factor of activated T cells, cytoplasmic, calcineurin-dependent 2<br>Nuclear factor of activated T cells 5, tonicity-responsive
RANTES	Chemokine (C-C motif) ligand 5<br>Chemokine (C-C motif) ligand 3<br>Chemokine (C-C motif) ligand 4
IL2	Interleukin 2
IL2RBT	Transcript of interleukin 2 recoptor, beta
IL2RB	Formation of the interleukin 2 low affinity receptor complex with interleukin 2 receptor, beta, gamma, also response to interleukin 15
IL2RAT	Transcript of interleukin 2 recoptor, alpha
IL2RA	Formation of the interleukin 2 high affinity receptor complex with the interleukin 2 receptor, alpha, beta, gamma
JAK	Janus kinase 1 (a protein tyrosine kinase)<br>Janus kinase 2 (a protein tyrosine kinase)<br>Janus kinase 3 (a protein tyrosine kinase, leukocyte)

SOCS	Suppressor of cytokine signaling 1<br>Suppressor of cytokine signaling 2<br>Suppressor of cytokine signaling 3
STAT3	Signal transducer and activator of transcription 3 (acute-phase response factor)<br>Signal transducer and activator of transcription 1, 91kDa<br>Signal transducer and activator of transcription 5A<br>Signal transducer and activator of transcription 5B
P27	Cyclin-dependent kinase inhibitor 2B (p15, inhibits CDK4)<br>Cyclin-dependent kinase inhibitor 1A (p21, Cip1)<br>Cyclin-dependent kinase inhibitor 1B (p27, Kip1)<br>Cyclin-dependent kinase inhibitor 1C (p57, Kip2)
Proliferation	N/A
TBET	T-box 21<br>Eomesodermin homolog (*Xenopus laevis*)
CREB	cAMP responsive element binding protein 1
IFNGT	Transcript of interferon, gamma
IFNG	Interferon, gamma
P2	N/A
GZMB	Granzyme B (granzyme 2, cytotoxic T-lymphocyte-associated serine esterase 1)<br>Perforin 1 (pore forming protein)
TPL2	Mitogen-activated protein kinase kinase kinase 8
TNF	Tumor necrosis factor (TNF superfamily, member 2)
TRADD	TNFRSF1A-associated via death domain
FasL	Fas ligand (TNF superfamily, member 6)
FasT	Transcript of Fas (TNF receptor superfamily, member 6)
Fas	Fas (TNF receptor superfamily, member 6)
sFas	Fas (TNF receptor superfamily, member 6), soluble Fas
Ceramide	Ceramide
DISC	Death inducing signaling complex
Caspase	Caspase 3, apoptosis-related cysteine peptidase<br>Caspase 8, apoptosis-related cysteine peptidase
FLIP	CASP8 and FADD-like apoptosis regulator
A20	Tumor necrosis factor, alpha-induced protein 3
BID	BH3 interacting domain death agonist<br>BCL2-like 11 (apoptosis facilitator)
IAP	Baculoviral IAP repeat-containing 2<br>Baculoviral IAP repeat-containing 3<br>Baculoviral IAP repeat-containing 4
BclxL	BCL2-like 1<br>B-cell CLL/lymphoma 2
MCL1	Myeloid cell leukemia sequence 1 (BCL2-related)
Apoptosis	N/A
GPCR	Endothelial differentiation, sphingolipid G protein-coupled receptor, 1<br>Endothelial differentiation, sphingolipid G protein-coupled receptor, 3<br>Endothelial differentiation, sphingolipid G protein-coupled receptor, 5<br>Endothelial differentiation, sphingolipid G protein-coupled receptor, 8
SMAD	SMAD family member 2<br>SMAD family member 3<br>SMAD family member 4
SPHK1	Sphingosine kinase 1
S1P	Sphingosine-1-phosphate

Reference

1. Zhang R, Shah MV, Yang J, Nyland SB, Liu X, et al. (2008) Network model of survival signaling in large granular lymphocyte leukemia. Proc Natl Acad Sci U S A 105: 16308-16313.
