# Supplementary material for: Dynamical and Structural Analysis of a T Cell Survival Network Identifies Novel Candidate Therapeutic Targets for Large Granular Lymphocyte Leukemia
Source: PLoS Comput Biol. 2011 Nov 10;7(11):e1002267. doi: 10.1371/journal.pcbi.1002267 (PMC3213185; doi:10.1371/journal.pcbi.1002267)
Supplement: Table S3 — Boolean rules governing the state of the 18-node sub-network depicted in Figure 2A . For simplicity, the nodes' states are represented by the node names. The symbol * indicates the future state of the marked node. (PDF) [file pcbi.1002267.s004.pdf]

Table S3. Boolean rules governing the state of the 18-node sub-network depicted in Figure 2A. For simplicity, the nodes' states are represented by the node names. The symbol * indicates the future state of the marked node.

**Node**	**Boolean rule**
CTLA4	CTLA4* = TCR AND NOT Apoptosis
TCR	TCR * = NOT (CTLA4 OR Apoptosis)
CREB	CREB * = IFNG AND NOT Apoptosis
IFNG	IFNG * = NOT (SMAD OR P2 OR Apoptosis)
P2	P2* = (IFNG OR P2) AND NOT Apoptosis
GPCR	GPCR * = S1P AND NOT Apoptosis
SMAD	SMAD * = GPCR AND NOT Apoptosis
Fas	Fas * = NOT (sFas OR Apoptosis)
sFas	sFas* = S1P AND NOT Apoptosis
Ceramide	Ceramide* = Fas AND NOT (S1P or Apoptosis)
DISC	DISC* = (Ceramide OR (Fas AND NOT FLIP)) AND NOT Apoptosis
Caspase	Caspase*= ((BID AND NOT IAP) OR DISC) AND NOT Apoptosis
FLIP	FLIP* = NOT (DISC OR Apoptosis)
BID	BID* = NOT (MCL1 OR Apoptosis)
IAP	IAP* = NOT (BID OR Apoptosis)
MCL1	MCL1* = NOT (DISC OR Apoptosis)
S1P	S1P* = NOT (Ceramide OR Apoptosis)
Apoptosis	Apoptosis* = Caspase OR Apoptosis
